# *PSEN1* Expression Identifies a Developmentally Distinct Favourable‐Prognosis State in SHH α Medulloblastoma

**DOI:** 10.1002/jdn.70178

**Published:** 2026-09-14

**Authors:** Julia Vanini, Amanda Thomaz, Marina Müller Lupatini, André T. Brunetto, Caroline Brunetto de Farias, Mariane Jaeger, Rafael Roesler

**Affiliations:** ^1^ Cancer and Neurobiology Laboratory, Experimental Research Center, Clinical Hospital (CPE‐HCPA) Federal University of Rio Grande do Sul Porto Alegre Brazil; ^2^ National Science and Technology Institute for Children's Cancer Biology and Pediatric Oncology—INCT BioOncoPed Porto Alegre Brazil; ^3^ Department of Biology Edge Hill University Ormskirk UK; ^4^ Children's Cancer Institute (ICI) Porto Alegre Brazil; ^5^ Department of Pharmacology, Institute for Basic Health Sciences Federal University of Rio Grande do Sul Porto Alegre Brazil; ^6^ Center for Biotechnology Federal University of Rio Grande do Sul Porto Alegre Brazil

**Keywords:** brain tumour, medulloblastoma, paediatric brain cancer, presenilin‐1, *PSEN1*, sonic hedgehog

## Abstract

**Background:**

Although *PSEN1* is best known for its role in Alzheimer's disease, it also regulates neural development and cerebellar morphogenesis. Medulloblastoma (MB) is the most common malignant paediatric brain tumour and arises from disrupted cerebellar developmental programmes. The clinical significance of *PSEN1* in MB remains unknown. We investigated the prognostic value and transcriptional correlates of *PSEN1* expression across molecular subgroups and subtypes of MB.

**Methods:**

Public bulk and single‐cell transcriptomic datasets were used to examine *PSEN1* expression, associations with overall survival (OS) and transcriptional correlates in MB. The SHH α‐associated transcriptional pattern was evaluated in an independent cohort, and *PSEN1* expression was further examined in the developing human cerebellum and across paediatric brain tumour types. Genes strongly correlated with *PSEN1* in SHH α MB were subjected to gene ontology (GO) enrichment analysis.

**Results:**

High *PSEN1* expression was consistently associated with significantly longer OS exclusively in SHH α MB. The *PSEN1*‐associated transcriptional pattern was reproduced in an independent SHH α cohort. *PSEN1* was expressed across developing cerebellar cell populations and paediatric brain tumour types, with MB showing intermediate expression among the tumour entities examined. In SHH α MB, *PSEN1* was associated with a coordinated transcriptional programme enriched for RNA homeostasis, intracellular membrane trafficking, protein quality control, lipid and calcium signalling and developmental pathways.

**Conclusions:**

High *PSEN1* expression identifies a favourable‐prognosis subset of SHH α MB and is associated with a distinct transcriptional programme related to endomembrane organization and cellular homeostasis rather than canonical SHH signalling. These findings suggest that *PSEN1* may mark a developmentally distinct tumour state and generate new hypotheses regarding subtype‐specific developmental programmes in MB.

## Graphical abstract

High *PSEN1* expression identifies a favourable‐prognosis state in SHH α medulloblastoma and is associated with a reproducible transcriptional programme related to cellular homeostasis and development. Its broad expression during cerebellar development and across paediatric brain tumours suggests that its prognostic significance reflects the specific molecular context of SHH α tumours.

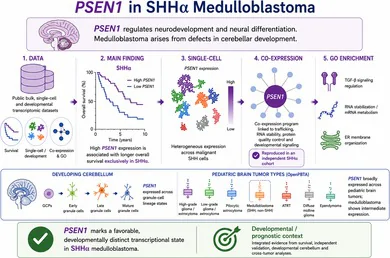

**Abbreviations**
AD: Alzheimer's disease
APP: amyloid precursor protein
ATRT: atypical teratoid/rhabdoid tumour
Aβ: amyloid‐β
BH: Benjamini–Hochberg
BP: biological process
CB: cerebellar tissue
CC: cellular component
EGA: European Genome‐Phenome Archive
EGL: external granule layer
EPN: ependymoma
ER: endoplasmic reticulum
ERAD: ER‐associated degradation
FDR: false discovery rate
GCP: granule cell precursor
GEO: Gene Expression Omnibus
GN: granule neuron
GO: gene ontology
HSD: honest significant difference
iPSC: induced pluripotent stem cells
MB: medulloblastoma
MF: molecular function
OpenPBTA: Open Pediatric Brain Tumor Atlas
OS: overall survival
PS1: presenilin‐1
RL: rhombic lip
scRNA‐seq: single‐cell RNA sequencing
SHH: sonic hedgehog
SNF: similarity network fusion
TGF‐β: transforming growth factor‐β
TPM: transcript‐per‐million
UMAP: Uniform Manifold Approximation and Projection
uRL: upper rhombic lip
WNT: wingless

## 1 Introduction

Paediatric brain tumours represent the most common solid malignancies in childhood and remain a leading cause of cancer‐related death among children. Medulloblastoma (MB) is the most frequently diagnosed malignant brain tumour, arising in the cerebellum and serving as a paradigm of how disrupted neurodevelopmental processes can drive oncogenesis. Current treatment relies on a combination of surgical resection, radiotherapy and chemotherapy, which has substantially improved survival rates over recent decades. Nevertheless, disease recurrence still occurs in a significant proportion of patients and is associated with a poor prognosis. Furthermore, treatment‐related toxicities often result in persistent neurocognitive, neurological and endocrine complications that can substantially affect long‐term quality of life (Northcott et al. 2019).

Our understanding of the complexity of MB biology has consistently advanced thanks to genomic approaches. It is now established that MB comprises four biologically distinct entities that present different clinical outcomes. These defined molecular subgroups are Wingless (WNT)‐activated, sonic hedgehog (SHH)‐activated, Group 3 and Group 4 MB (Taylor et al. 2012; Northcott et al. 2017; Juraschka and Taylor 2019).

Given the substantial heterogeneity within each subgroup, they are currently further subdivided into subtypes. Through the Medulloblastoma Advanced Genomics International Consortium, Cavalli et al. (2017) assembled a cohort of 763 primary MB samples and applied similarity network fusion (SNF) and spectral clustering within each of the four subgroups across the cohort to integrate gene expression and DNA methylation profiling and generate a genome‐wide profile. This led to the identification and characterization of 12 subtypes that show distinct somatic copy‐number aberrations and clinical features, two of them within the WNT subgroup, four in SHH, three in Group 3 and three in Group 4 tumours.

The *PSEN1* gene encodes presenilin‐1 (PS1), the catalytic subunit of the γ‐secretase complex, a multiprotein protease responsible for cleaving numerous transmembrane substrates, notably amyloid precursor protein (APP). Through its role in APP processing, PS1 contributes to the generation of amyloid‐β (Aβ) peptides. *PSEN1* is widely regarded as a major Alzheimer's disease (AD) gene, as pathogenic *PSEN1* mutations represent the most common cause of autosomal dominant early‐onset familial AD (Sherrington et al. 1995). However, it also regulates neural development and differentiation. Lack of *PSEN1* leads to early differentiation of neural progenitor cells, leading to abnormal neuronal migration and disorganization of the laminar architecture in the developing brain (Handler et al. 2000). *PSEN1* mutations associated with familial AD result in premature neurogenesis in brain organoids derived from human induced pluripotent stem cells (iPSCs) (Arber et al. 2021). In the cerebellum, *PSEN1* inactivation disrupts the spread of granule cell precursors (GCPs) to form the external granule layer (EGL) during development (Louvi et al. 2004).

Previous studies have not examined a possible role for *PSEN1* or PS1 in MB. In this study, we used the Cavalli cohort, first screening each MB subtype for possible associations between *PSEN1* expression levels and patient outcome indicated by overall survival (OS). Of all 12 subtypes, only SHH α had a consistent statistically significant association with better OS. Among the four SHH MB subtypes (SHH α, SHH β, SHH γ and SHH δ), SHH α is biologically the most genomically unstable and clinically aggressive subtype. Afflicting mostly children aged 3–16 years, SHH α tumours are characterized by amplifications of *MYCN*, *GLI2* and *YAP1*, loss of 9q, 10q and 17p and a high rate of *TP53* mutations that define one of the worst prognoses among MB subtypes (Northcott et al. 2012; Cavalli et al. 2017; Coltin et al. 2021; Skowron et al. 2021; Gold et al. 2024). We then aimed to explore the expression and co‐expression structure of *PSEN1* in SHH α.

## 2 Methods

### 2.1 *PSEN1* Gene Expression Across MB Subgroups and Subtypes

The Pomeroy dataset established by Cho et al. (2011) (accession number GSE202043) was used to compare *PSEN1* expression in MB tumours (*n* = 194) versus nontumoural cerebellar tissue (CB, *n* = 11). *PSEN1* mRNA expression across MB molecular subgroups and subtypes was evaluated using the GlioVis data portal (https://gliovis.bioinfo.cnio.es/) (Bowman et al. 2017). Analyses were performed using the Cavalli MB dataset (Cavalli et al. 2017; accession number GSE85217), which comprises 763 primary MB samples classified into the four distinct molecular subgroups and 12 molecular subtypes, and the platform's built‐in plotting tools were used to visualize *PSEN1* expression distributions among molecular subgroups and subtype‐specific cohorts. The comparison between MB tumours and CB was performed with a two‐tailed Mann–Whitney *U* test. Comparisons among subgroups and subtypes were performed using Tukey's honest significant difference (HSD) tests.

### 2.2 *PSEN1* Expression in MB Single‐Cell RNA Sequencing Data

*PSEN1* expression at the single‐cell level was evaluated using MB single‐cell RNA sequencing (scRNA‐seq) data from the dataset GSE156053 (Riemondy et al. 2022), available through the Gene Expression Omnibus (GEO) repository. To investigate the distribution of *PSEN1* expression across MB cellular populations and SHH molecular subtypes, data were explored using the Pediatric Neuro‐oncology Cell Atlas interactive platform (https://www.pneuroonccellatlas.org/). Uniform Manifold Approximation and Projection (UMAP) embeddings were used to visualize *PSEN1* expression patterns at single‐cell resolution across the different MB subgroups.

### 2.3 Survival Analysis

Associations between gene expression and OS were evaluated using the R2 Genomics Analysis and Visualization Platform (https://hgserver1.amc.nl/). Kaplan–Meier survival analyses were performed using the platform's default settings. Patients were stratified into low‐ and high‐expression groups according to two approaches: (1) the median expression value and (2) the optimal expression cut‐off determined by the R2 scan procedure, which systematically evaluates potential cut‐off values and selects the threshold yielding the strongest separation between survival curves.

Statistical significance was assessed using the log‐rank test. For analyses based on the optimal scan cut‐off, both the raw log‐rank *p*‐values and the multiple‐testing‐adjusted *p*‐values provided by the platform were recorded, including Bonferroni‐corrected and Benjamini–Hochberg (BH) false discovery rate (FDR)‐adjusted *p*‐values. Results obtained using the median cut‐off and scan cut‐off approaches were compared to evaluate the robustness and consistency of survival associations across different patient stratification strategies.

### 2.4 Clinical Annotation Heatmap

To explore the relationship between *PSEN1* expression and clinicopathological features within the SHH α MB subtype, a heatmap was generated using the R2 Genomics Analysis and Visualization Platform. Samples were ordered according to *PSEN1* expression levels, and corresponding clinical annotations were displayed alongside expression values. The clinical variables examined included age group, vital status (alive/deceased), metastatic status, disease status and histological subtype. This analysis was performed for exploratory and visualization purposes to assess potential patterns linking *PSEN1* expression with clinical characteristics. No formal statistical testing was performed for the heatmap analysis, which was intended to provide a visual overview of the distribution of clinical features across *PSEN1* expression levels.

### 2.5 *PSEN1* Co‐Expression Structure

Genes co‐expressed with *PSEN1* were identified using Spearman correlation analysis in SHH α MB samples. Genes exhibiting strong positive correlations with *PSEN1* (*r* ≥ 0.62) were ranked, and the 26 most strongly correlated genes were selected for visualization. A strong correlation was defined as *r* between 0.62 and 0.88 with a statistically significant *p* value, and a very strong correlation was defined as *r* > 0.89 with a statistically significant *p* value. These thresholds and ranges were chosen to retain genes showing consistently strong positive associations with *PSEN1* while maintaining a sufficiently large gene set for meaningful functional enrichment analysis. Correlation coefficients in this range are commonly interpreted as representing strong positive relationships in biomedical research. A heatmap displaying the expression profiles of *PSEN1* and these genes across tumour samples was generated in R (version 4.5.3). Expression values were visualized using unsupervised hierarchical clustering of genes and samples to identify patterns of coordinated transcriptional activity associated with *PSEN1* expression.

### 2.6 Functional Enrichment Analysis

To investigate biological processes (BPs) associated with *PSEN1* expression in SHH α MB, gene co‐expression analysis was performed using the R2 Genomics Analysis and Visualization Platform in the Cavalli cohort. Analyses were restricted to the SHH α molecular subtype. Genes exhibiting strong to very strong positive correlations with *PSEN1* expression (Spearman correlation coefficient *r* ≥ 0.62) were selected, yielding a set of 26 strongly co‐expressed genes. This threshold was selected based on commonly used interpretations of correlation strength in biomedical research, in which coefficients above approximately 0.60 are generally considered strong positive correlations and approach the range often described as very strong correlations. The threshold was chosen to prioritize biologically meaningful associations while maintaining adequate sensitivity for downstream analyses of co‐expression networks.

Functional enrichment analysis of the resulting gene set was performed using Enrichr (https://maayanlab.cloud/Enrichr/). Gene ontology enrichment was assessed across the BP, cellular component (CC) and molecular function (MF) categories. Enriched terms were ranked according to the significance metrics provided by the platform, and terms with adjusted *p*‐values < 0.05 were considered statistically significant.

### 2.7 Independent Validation of *PSEN1* Expression and Co‐Expression Patterns

To independently assess the molecular findings obtained in the Cavalli cohort, gene expression data from an additional MB dataset were analysed using the harmonized expression matrix reported by Arora et al. (2026). Molecular subtype annotations for SHH MB were obtained from the Skowron et al. (2021) cohort. Sample identifiers were matched between datasets, yielding 196 SHH tumours with available molecular subtype classification, comprising 50 SHH α, 42 SHH β, 32 SHH γ and 72 SHH δ tumours. *PSEN1* expression was compared across SHH molecular subtypes using the Kruskal–Wallis test, followed by pairwise Wilcoxon rank‐sum tests with BH correction for multiple comparisons.

To independently evaluate the *PSEN1*‐associated transcriptional pattern identified in the Cavalli SHH α cohort, analyses were restricted to the 50 independently classified SHH α tumours. Pearson correlation coefficients between *PSEN1* expression and genome‐wide gene expression were calculated. Of the 26 genes previously identified as strongly positively correlated with *PSEN1* in Cavalli SHH α tumours, 22 were represented in the independent expression matrix and were therefore evaluated as a prespecified validation gene set. Enrichment of these genes among the 100 genes showing the strongest positive correlations with *PSEN1* was assessed using a hypergeometric test. In addition, an empirical permutation analysis was performed by randomly sampling 100,000 sets of 22 genes from the genome‐wide background distribution and comparing their median *PSEN1* correlation coefficients with that of the prespecified validation set. All analyses were performed in R (version 4.5.3) using RStudio (version 2026.08.1+195; Posit Software, PBC).

### 2.8 Developing Human Cerebellum Single‐Nucleus Transcriptomic Analyses

*PSEN1* expression during normal human cerebellar development was examined in two independent single‐nucleus RNA‐sequencing datasets. First, we analysed the developing human cerebellum atlas reported by Aldinger et al. (2021), comprising 69,174 nuclei sampled across 9–21 postfertilization weeks. Cell‐type and developmental‐stage annotations provided with the dataset were retained. *PSEN1* expression was summarized across annotated cell populations as mean expression and the percentage of nuclei with detectable expression. Expression was also examined across developmental stages.

To specifically assess the granule cell developmental lineage, nuclei annotated as rhombic lip (RL), GCP and granule neuron (GN) were analysed. Because nuclei obtained from the same donor are not independent biological replicates, statistical comparisons among these lineage states were performed using donor‐level mean *PSEN1* expression. Donors represented in all three states were included in the paired analysis, and differences among RL, GCP and GN were assessed using the Friedman test.

As an independent validation, *PSEN1* expression was additionally examined in the developing human cerebellum single‐nucleus transcriptomic atlas reported by Sepp et al. (2024). Analyses were restricted to cells assigned by the authors to the RL/EGL developmental lineage. *PSEN1* expression was summarized across the author‐defined developmental states as mean expression and percentage of nuclei with detectable expression. Processed single‐nucleus datasets and accompanying cell annotations were obtained through CELLxGENE (https://cellxgene.cziscience.com/). All analyses were performed in R (version 4.5.3) using RStudio (version 2026.08.1+195; Posit Software, PBC).

### 2.9 *PSEN1* Expression Across Paediatric Brain Tumour Types

*PSEN1* expression across paediatric brain tumour types was examined using RNA‐sequencing data from the Open Pediatric Brain Tumor Atlas (OpenPBTA; https://github.com/AlexsLemonade/OpenPBTA‐analysis; Shapiro et al. 2023). Gene‐level expression data were obtained from the stranded RNA‐sequencing RSEM transcript‐per‐million (TPM) matrix from OpenPBTA release v23 (release‐v23‐20230115) and matched to harmonized tumour annotations using Kids First Biospecimen identifiers. The analysis included seven paediatric brain tumour groups represented by at least 30 samples in the expression dataset: high‐grade glioma/astrocytoma (*n* = 93), low‐grade glioma/astrocytoma (*n* = 86), pilocytic astrocytoma (*n* = 131), MB (*n* = 121), atypical teratoid/rhabdoid tumour (ATRT; *n* = 30), diffuse midline glioma (*n* = 34) and ependymoma (EPN, *n* = 93). Differences in *PSEN1* expression across tumour groups were assessed using the Kruskal–Wallis test. Pairwise comparisons were subsequently performed using Wilcoxon rank‐sum tests with BH correction for multiple comparisons. All analyses were performed in R (version 4.5.3) using RStudio (version 2026.08.1+195; Posit Software, PBC).

## 3 Results

### 3.1 High *PSEN1* Expression Is Selectively Associated With Better Survival in SHH α MB

**FIGURE 1:**
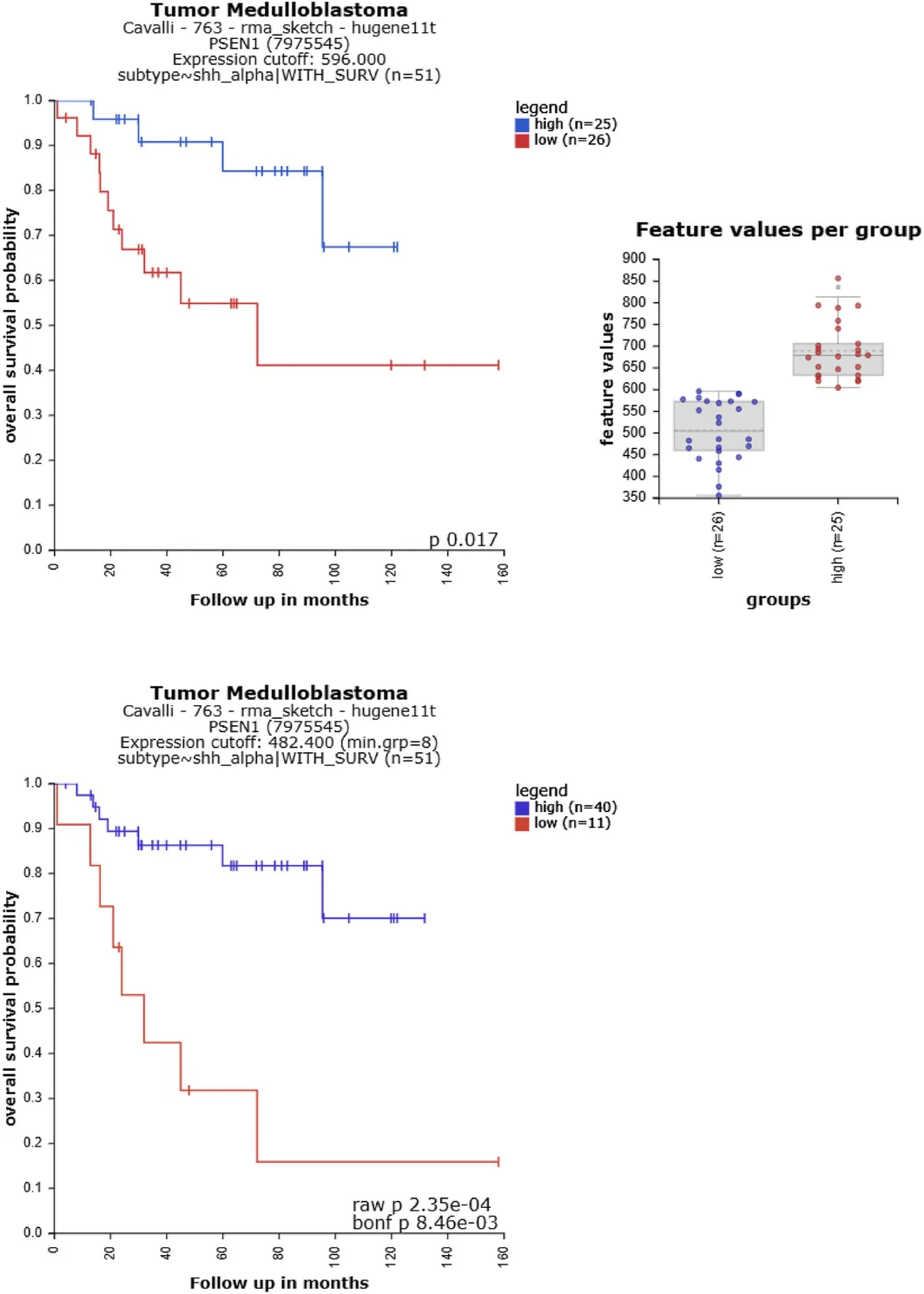
Kaplan–Meier analyses of OS according to *PSEN1* gene expression levels in SHH α MB tumours. Survival analyses were performed using the R2 Genomics Analysis and Visualization Platform. Upper panels, results using the raw *p* value with median cut‐off. Lower panel, raw and Bonferroni‐adjusted *p* values using optimal scan cut‐off; *p* values are shown in the panels and Table S1.

We first screened *PSEN1* expression separately in all 12 MB subtypes to determine associations with patient survival, using four complementary statistical approaches to determine whether associations were consistent across different methods of patient stratification. Across four different statistical criteria for significance, namely, showing the raw *p*‐value for analyses using the median as cut‐off for separating low versus high expression; the Bonferroni‐adjusted *p*‐value with the R2 scan tool cut‐off; the raw *p*‐value with the scan cut‐off; and FDR‐adjusted *p*‐value obtained using the BH method to adjust for multiple comparisons, with the scan cut‐off, the only subtype showing significant associations between *PSEN1* expression and survival in all statistical methods was SHH α, where *PSEN1* transcription showed a consistent correlation with longer OS (Figure 1, Table S1).

### 3.2 *PSEN1* Expression Levels in MB Tumours Classified According to Molecular Subgroup and Subtype

**FIGURE 2:**
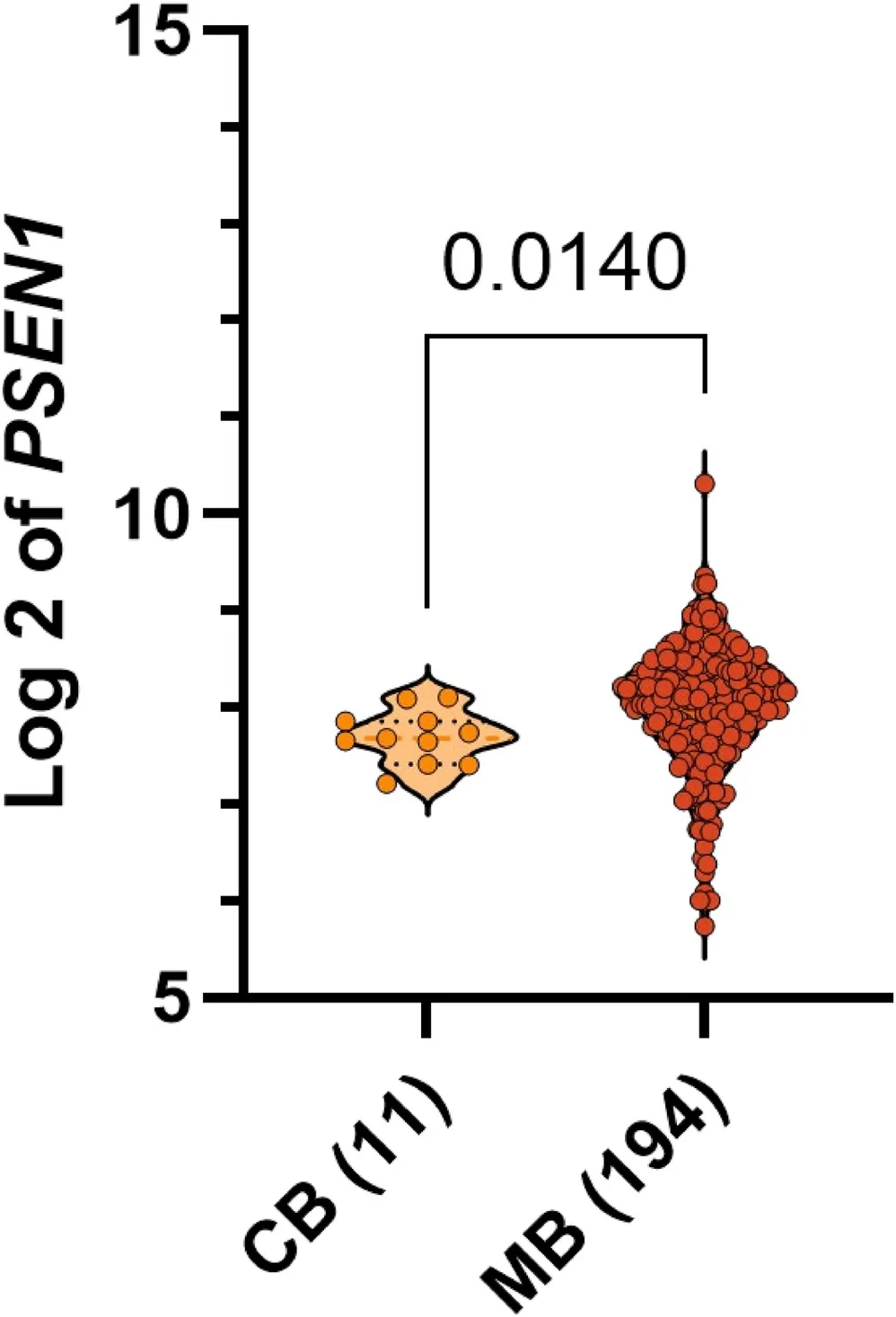
*PSEN1* expression in MB tumours and nontumoural cerebellar tissue (CB). Transcription levels were compared between CB (*n* = 11) and MB samples (*n* = 194). Data were obtained from the Pomeroy dataset (GSE202043) described by Cho et al. (2011). Each point represents an individual sample. Statistical significance was assessed using a two‐tailed Mann–Whitney *U* test, with the *p* value indicated above the comparison. The bracket denotes the statistically significant difference between groups. Data are presented as log_2_‐transformed *PSEN1* mRNA level values.

**FIGURE 3:**
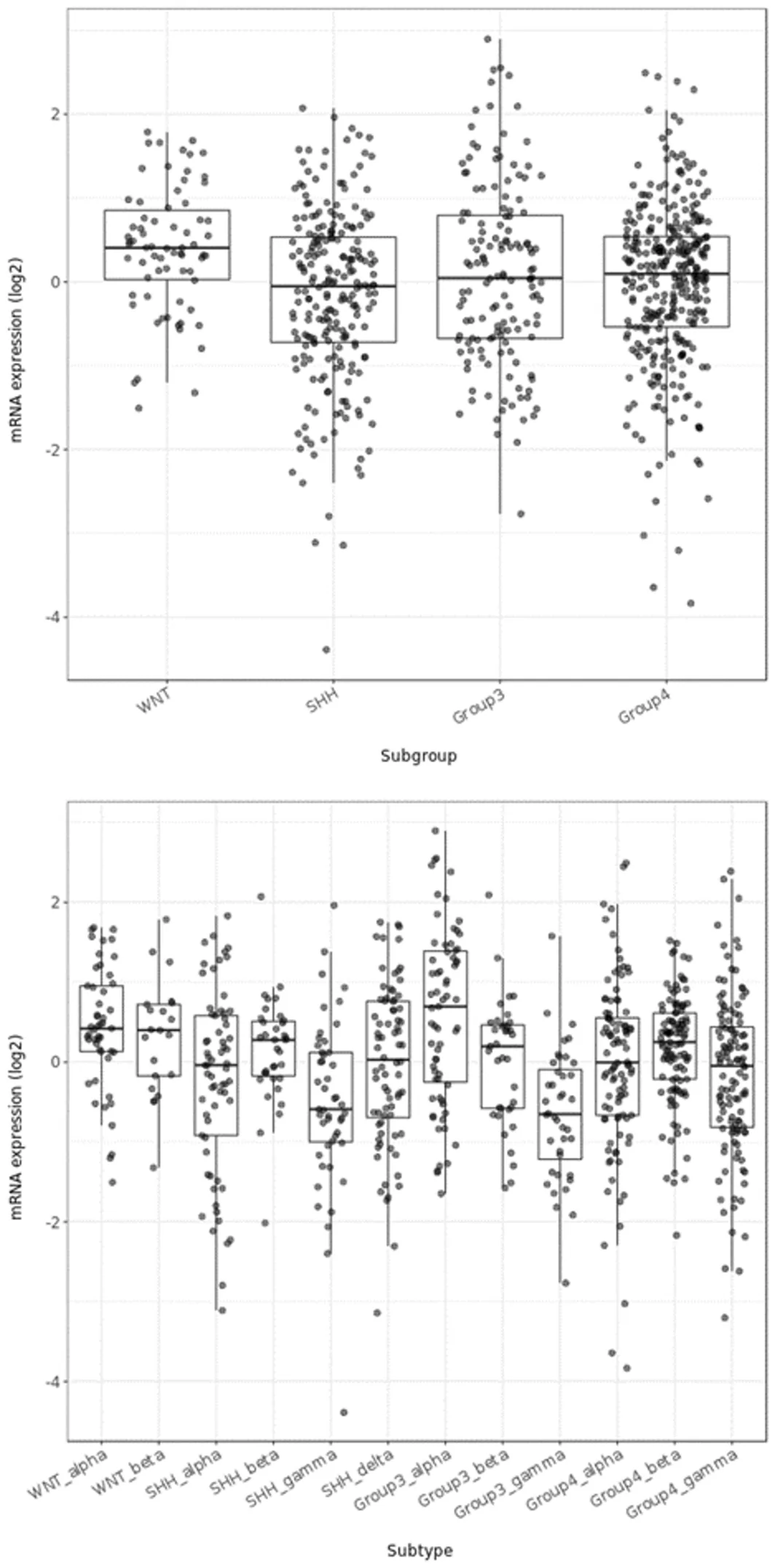
*PSEN1* expression across MB molecular subgroups and subtypes in the Cavalli cohort. Upper panel, comparison among the four molecular subgroups. Lower panel, comparison among the 12 molecular subtypes. Statistical analyses were performed using the Tukey's honest significant difference (HSD) test, and *p* values are shown in Tables S2 and S3.

*PSEN1* transcription levels were significantly higher in MB tumours in comparison with nontumoural CB (Figure 2). Among molecular subgroups, SHH tumours exhibited significantly lower *PSEN1* expression than WNT tumours, whereas WNT tumours showed significantly higher expression than Group 4 tumours (Figure 3, Table S2). No other subgroup comparisons reached statistical significance. At the subtype level, SHH α tumours displayed significantly lower *PSEN1* expression than WNT‐α and Group 3‐α tumours (Figure 3, Table S3). No significant differences were observed between SHH α and the remaining subtypes.

**FIGURE 4:**
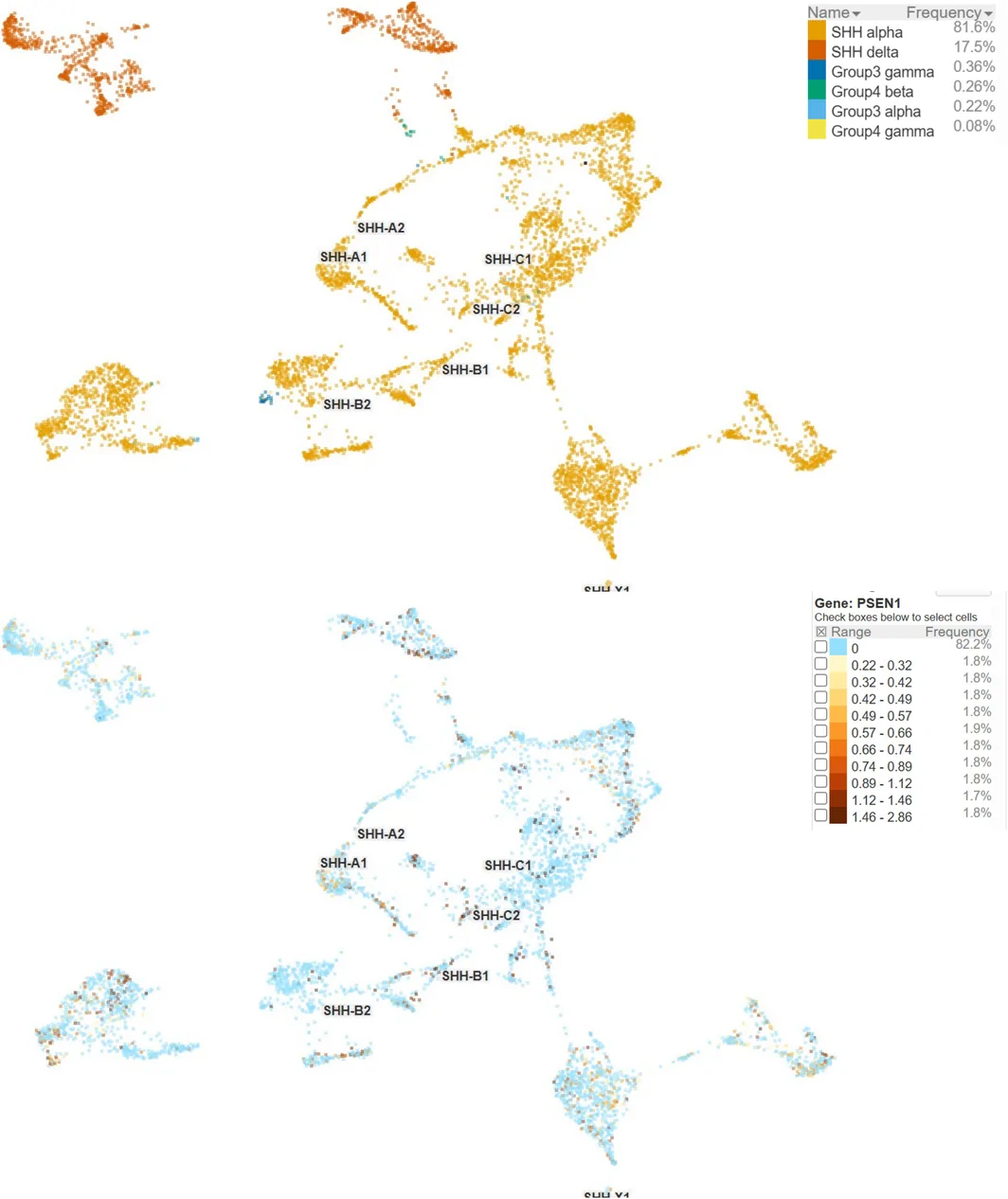
UMAP visualization of *PSEN1* expression in single‐cell RNA sequencing data from SHH MB samples. *PSEN1*‐positive cells are distributed throughout malignant SHH tumour populations and subtypes rather than being confined to a discrete cellular cluster. Labels refer to single‐cell transcriptional states identified by clustering individual malignant cells across SHH tumours (Riemondy et al. 2022).

To investigate *PSEN1* expression at the single‐cell level, scRNA‐seq data from SHH MB samples were analysed. UMAP visualization demonstrated heterogeneous *PSEN1* expression across malignant cell populations, with *PSEN1*‐positive cells distributed throughout the SHH tumour cell landscape rather than being restricted to a discrete cellular cluster (Figure 4). Within the SHH α samples from the Cavalli cohort (*n* = 65), *PSEN1* expression scores were further visualized according to age group, survival status, histological subtype and metastatic status (Figure S1).

### 3.3 *PSEN1* Co‐Expression Network and Functional Enrichment Analysis in SHH α MB

**FIGURE 5:**
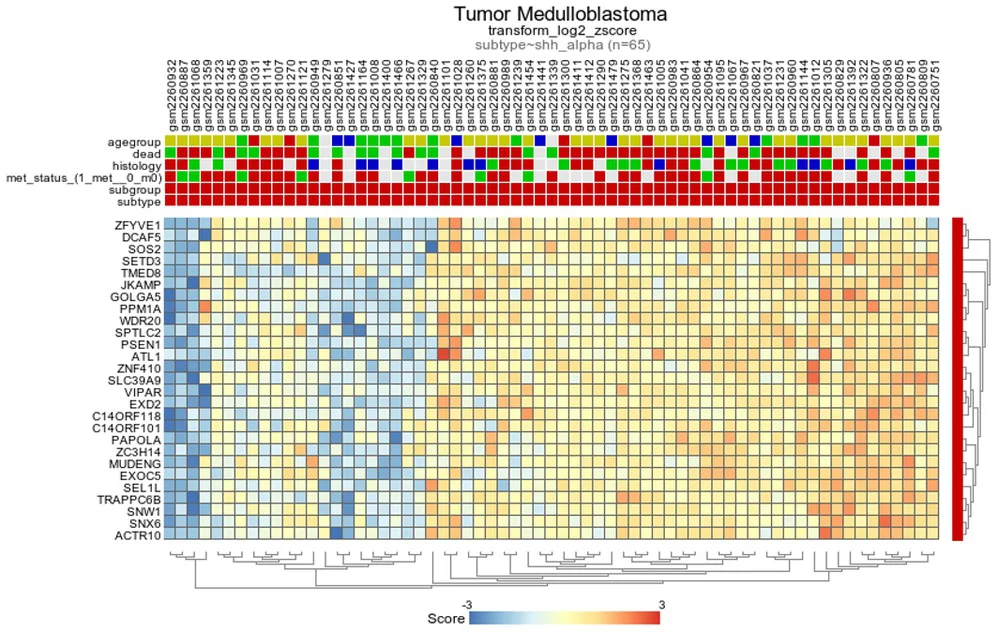
Heatmap showing coordinated expression of *PSEN1* and the 26 top genes displaying the strongest positive correlations with *PSEN1* in SHH α MB. Expression values are represented as log_2_‐transformed z‐scores. Both genes and tumour samples were hierarchically clustered using unsupervised clustering.

To explore candidate BPs associated with *PSEN1* expression in SHH α MB, co‐expression analyses were performed using transcriptomic data from the Cavalli cohort. Twenty‐six genes exhibited strong or very strong positive correlations with *PSEN1* expression and were selected for subsequent analyses. The strongest correlations were observed for *SPTLC2*, *ATL1*, *EXD2*, *ZNF410*, *PAPOLA*, *SOS2*, *EXOC5*, *VIPAR*, *SEL1L*, *SLC39A9*, *MUDENG*, *C14ORF118*, *TMED8*, *SETD3*, *WDR20*, *C14ORF101*, *SNX6*, *DCAF5*, *GOLGA5*, *PPM1A*, *ACTR10*, *TRAPPC6B*, *SNW1*, *ZFYVE1*, *ZC3H14* and *JKAMP* (Table S4). Hierarchical clustering demonstrated a coordinated expression pattern among *PSEN1* and its co‐expressed genes across SHH α tumours (Figure 5).

**FIGURE 6:**
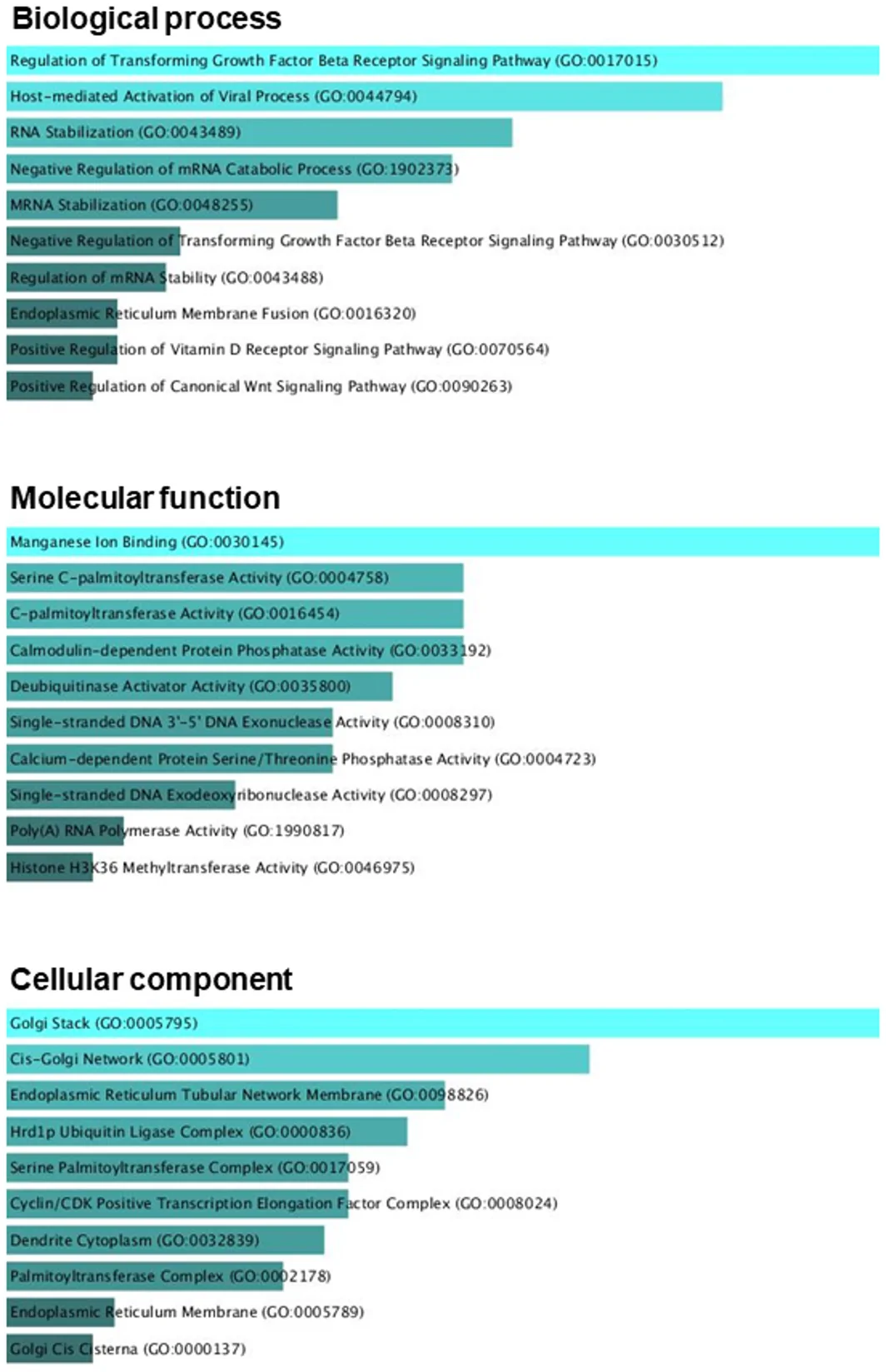
Gene ontology enrichment analysis of the 26 genes most strongly correlated with *PSEN1* in SHH α MB. Biological process (BP), cellular component (CC) and molecular function (MF) analyses demonstrate enrichment for RNA homeostasis, intracellular membrane trafficking, protein quality control, developmental signalling pathways and neuronal membrane‐associated functions.

Gene ontology enrichment analysis of the *PSEN1* co‐expression signature identified significant enrichment of terms related to RNA homeostasis, protein processing, intracellular trafficking and membrane‐associated cellular compartments. Within the BP category, the most significantly enriched terms included regulation of transforming growth factor β receptor signalling pathway, RNA stabilization, mRNA stabilization, negative regulation of mRNA catabolic process and regulation of mRNA stability. CC analysis identified enrichment of structures associated with intracellular membrane trafficking and protein quality control, including the Hrd1 ubiquitin ligase ERAD‐L complex. MF analysis revealed enrichment of activities related to sphingolipid biosynthesis, calcium/calmodulin‐dependent phosphatase signalling, ubiquitin‐mediated protein quality control, RNA processing, DNA repair and epigenetic regulation. These findings suggest that the *PSEN1*‐associated transcriptional programme is linked to neuronal membrane homeostasis, intracellular calcium signalling and maintenance of differentiated cellular functions rather than to proliferative pathways (Figure 6).

Together, these findings demonstrate that elevated *PSEN1* expression in SHH α MB is associated with a transcriptional programme enriched for genes involved in RNA regulation, intracellular membrane systems, protein quality control and developmental signalling pathways.

### 3.4 Independent Validation of *PSEN1*‐Associated Molecular Features in SHH MB

We next examined *PSEN1* expression and its associated transcriptional pattern in an independent cohort of molecularly characterized SHH MB (Arora et al. 2026; Skowron et al. 2021). A total of 196 tumours could be assigned to SHH molecular subtypes, including 50 SHH α, 42 SHH β, 32 SHH γ and 72 SHH δ tumours. *PSEN1* expression differed modestly across the four SHH subtypes (*p* = 0.041). Median expression values were 3.220, 3.405, 2.960 and 3.115 in SHH α, SHH β, SHH γ and SHH δ, respectively. In pairwise analyses, only the difference between SHH β and SHH γ remained significant after BH correction (adjusted *p* = 0.026), whereas SHH α did not differ significantly from the other individual subtypes (Figure S2). Thus, consistent with the Cavalli dataset analysis, the distinctive association of *PSEN1* with SHH α biology does not appear to result simply from selectively elevated *PSEN1* expression in this subtype.

We then asked whether the *PSEN1*‐associated transcriptional pattern identified in Cavalli SHH α tumours could be reproduced in the independent set of 50 SHH α tumours. Of the 26 genes comprising the Cavalli‐derived *PSEN1*‐associated gene set, 22 were represented in the independent expression matrix. Remarkably, all 22 genes showed positive correlations with *PSEN1*, with Pearson correlation coefficients ranging from 0.674 to 0.944 and a median correlation of 0.880 (Figure S3). Fifteen of the 22 genes ranked among the 100 genes most strongly correlated with *PSEN1* genome‐wide, representing significant enrichment relative to chance (hypergeometric *p* = 1.29 × 10^−29^). Moreover, the median correlation of the prespecified 22‐gene set exceeded that obtained for all 100,000 randomly sampled gene sets of equal size (empirical *p* < 1 × 10^−5^). These findings independently support the reproducibility of the *PSEN1*‐associated transcriptional pattern observed in SHH α MB.

### 3.5 *PSEN1* Is Expressed Across Cell Populations of the Developing Human Cerebellum

**FIGURE 7:**
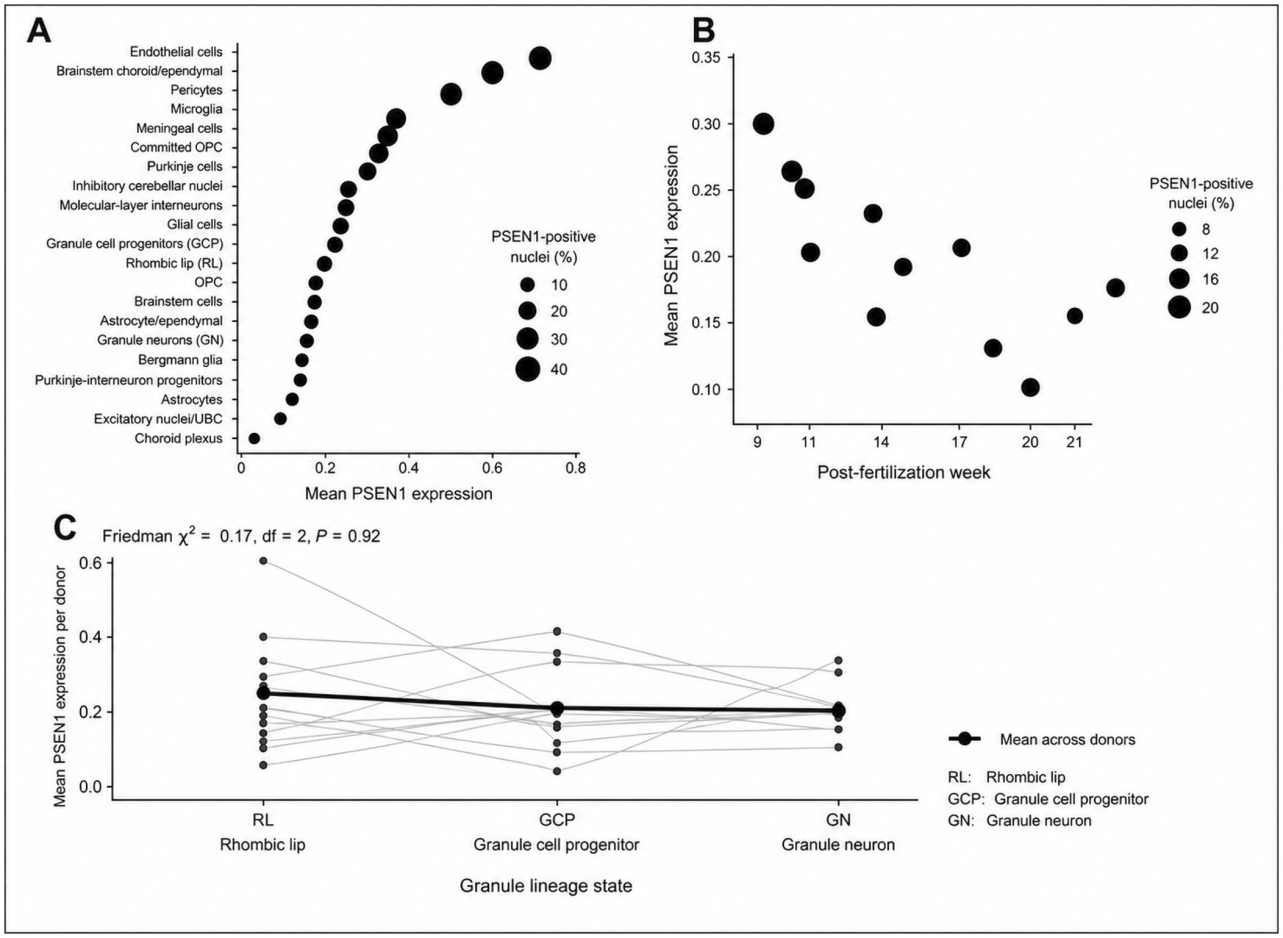
*PSEN1* expression in the developing human cerebellum. Single‐nucleus RNA‐sequencing data from the developing human cerebellum were analysed using the dataset of Aldinger et al. (2021). (A) *PSEN1* expression across 21 annotated cerebellar cell populations. Dot position indicates mean *PSEN1* expression, and dot size indicates the percentage of nuclei with detectable *PSEN1* expression. (B) Mean *PSEN1* expression across postfertilization developmental stages. Dot size indicates the percentage of *PSEN1*‐positive nuclei. Developmental‐stage values are presented descriptively because developmental age is associated with differences in donor and cellular composition. (C) Donor‐level mean *PSEN1* expression across the RL–GCP–GN developmental lineage. Thin lines connect values from the same donor, whereas the thick line and large points indicate mean expression across donors. No significant difference was observed among lineage states (Friedman χ^2^ = 0.17, df = 2, *p* = 0.92).

To characterize the developmental context of *PSEN1* expression, we first analysed a single‐nucleus transcriptomic atlas comprising 69,174 nuclei from the developing human cerebellum across 9–21 postfertilization weeks (Aldinger et al. 2021). *PSEN1* was detected across multiple cerebellar cell populations, with the highest mean expression observed in endothelial cells, brainstem choroid/ependymal cells and pericytes (Figure 7A), indicating that its expression is not restricted to a specific neuronal lineage. Importantly, *PSEN1* was detected throughout the RL–granule cell lineage, including RL cells (mean expression, 0.211; 15.2% *PSEN1*‐positive nuclei), GCPs (0.217; 14.0%) and GNs (0.133; 10.1%). *PSEN1* expression was detectable throughout the sampled prenatal developmental period, with descriptively lower mean expression at several later developmental stages (Figure 7B). Because developmental stage is associated with differences in donor and cellular composition, this pattern was considered descriptive rather than evidence of longitudinal developmental regulation.

We next compared *PSEN1* expression across the RL–GCP–GN lineage using donor‐level mean expression to account for the nonindependence of nuclei from the same individual. No significant difference was observed among the three lineage states (Friedman χ^2^ = 0.17, df = 2, *p* = 0.92; Figure 7C), indicating that *PSEN1* is expressed across this developmental trajectory without evidence of preferential expression at a particular granule‐lineage state.

We then examined an independent developing human cerebellum single‐nucleus dataset from Sepp et al. (2024). *PSEN1* expression was again detected across author‐defined RL/EGL developmental populations (Figure S4). In particular, *PSEN1* was readily detected in GCPs (*n* = 2714; mean expression = 0.237; 18.4% positive nuclei), early differentiating granule cells (GC_diff_1; *n* = 4251; mean = 0.231; 18.3% positive) and defined granule cells (GC defined; *n* = 47,116; mean = 0.222; 17.4% positive). Expression was also detected in the GC_diff_2 state (*n* = 14,715; mean = 0.175; 14.8% positive). Thus, an independent developmental cerebellum dataset confirmed *PSEN1* expression across granule cell progenitor and differentiating granule cell states.

### 3.6 *PSEN1* Is Expressed Across Different Paediatric Brain Tumour Types

**FIGURE 8:**
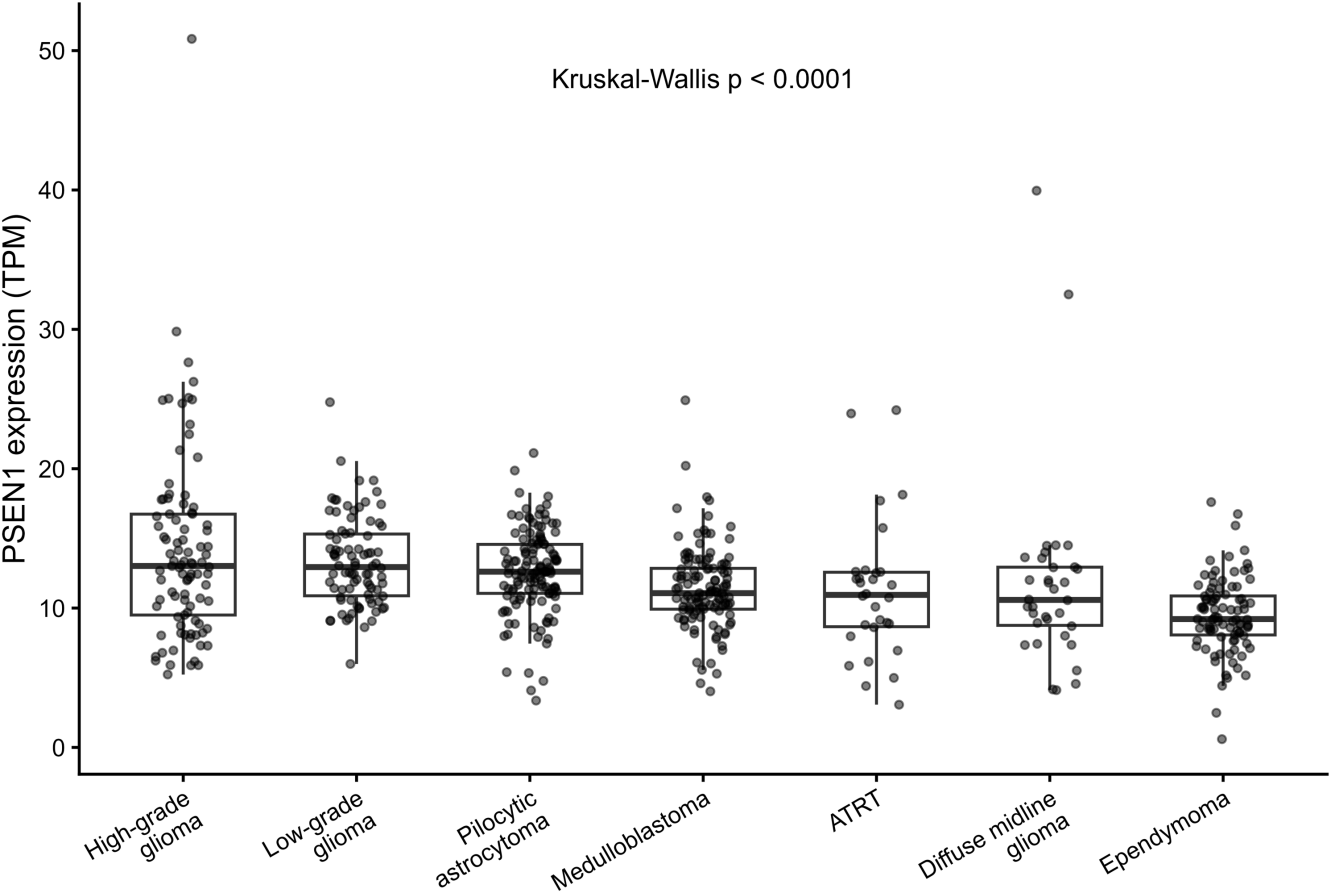
*PSEN1* expression across paediatric brain tumour types. *PSEN1* expression was examined using RNA‐sequencing data from the OpenPBTA, including high‐grade glioma/astrocytoma (*n* = 93), low‐grade glioma/astrocytoma (*n* = 86), pilocytic astrocytoma (*n* = 131), MB (*n* = 121), ATRT (*n* = 30), diffuse midline glioma (*n* = 34) and EPN (*n* = 93). Expression levels are shown as TPM. Boxes represent the interquartile range, with the horizontal line indicating the median; individual points represent tumour samples. Differences among tumour types were assessed using the Kruskal–Wallis test (*p* < 0.0001). Pairwise comparisons were performed using Wilcoxon rank‐sum tests with BH correction for multiple comparisons, and the results for these comparisons are described in the main text.

To place *PSEN1* expression in MB within the broader context of paediatric brain tumours, we examined *PSEN1* transcript levels across seven tumour groups in the OpenPBTA dataset (Shapiro et al. 2023). The results are shown in Figure 8. *PSEN1* was expressed across all tumour types examined, but its expression differed significantly among tumour groups (Kruskal–Wallis χ^2^ = 87.74, df = 6, *p* < 0.0001). Median *PSEN1* expression was 13.0 TPM in high‐grade glioma/astrocytoma, 12.9 TPM in low‐grade glioma/astrocytoma, 12.6 TPM in pilocytic astrocytoma, 11.1 TPM in MB, 10.9 TPM in ATRT, 10.6 TPM in diffuse midline glioma and 9.21 TPM in EPN. Pairwise Wilcoxon rank‐sum tests with BH correction showed that *PSEN1* expression in MB was significantly lower than in high‐grade glioma/astrocytoma (adjusted *p* = 0.0069), low‐grade glioma/astrocytoma (adjusted *p* = 9.12 × 10^−5^) and pilocytic astrocytoma (adjusted *p* = 4.70 × 10^−4^), and significantly higher than in EPN (adjusted *p* = 2.15 × 10^−6^). No significant differences were observed between MB and either ATRT or diffuse midline glioma (adjusted *p* = 0.434 for both comparisons). Thus, *PSEN1* is broadly expressed across paediatric brain tumours, with MB displaying intermediate expression levels among the tumour types examined.

## 4 Discussion

Characterizing biological and clinical differences among MB subtypes is critical for improving risk stratification. SHH MB is thought to arise predominantly from GCPs located in the EGL of the developing cerebellum. These cells are derived from the upper RL (uRL) and are committed to a glutamatergic excitatory neuronal lineage. Tumourigenesis is typically initiated by activating alterations in the SHH signalling pathway, including mutations in *PTCH1*, *SMO* and *SUFU*, whereas *MYCN* amplification is associated with a more aggressive clinical course and poorer outcome (Oliver et al. 2005; Yang et al. 2008; Gibson et al. 2010; Vladoiu et al. 2019; Gold et al. 2024; Falcón et al. 2026). Within the SHH subgroup, SHH α tumours are distinguished by marked chromosomal instability, extensive copy‐number alterations, recurrent whole‐chromosome arm gains and losses, frequent focal amplifications and an unfavourable prognosis (Cavalli et al. 2017).

We have previously shown that several genes involved in neurodevelopment and neural plasticity can display different and often opposite patterns of association with patient survival in different molecular subgroups of MB (de Araújo et al. 2023; Fratini et al. 2023; Monteiro et al. 2024; Coelho et al. 2025; Saciloto et al. 2026). The present study highlights the importance of further investigating molecular targets and candidate prognostic markers at the subtype level within each molecular subgroup.

The proliferation of GCPs in the developing cerebellum is regulated by SHH signalling (Wallace 1999; Wechsler‐Reya and Scott 1999). PS1 can enhance SHH expression and functionally interact with SHH to impair neurogenesis and promote cell proliferation and apoptosis (Paganelli et al. 2001). This evidence supports the possibility that PS1 is a direct modulator of SHH signalling, which could result in influences on SHH α MB aggressiveness. In neuronal progenitors and human iPSCs, mutations in *PSEN1* result in a premature differentiation phenotype, through a mechanism likely involving Notch signalling inhibition (Handler et al. 2000; Arber et al. 2021), suggesting that *PSEN1* contributes to the preservation of immature neural states. At first glance, our finding that elevated *PSEN1* expression is associated with improved survival appears counterintuitive, as stem cell‐associated programmes are often linked to tumour aggressiveness. However, single‐cell studies have shown that MB contains cells spanning multiple developmental states, and the most aggressive populations are not always the least differentiated (Hovestadt et al. 2019; Riemondy et al. 2022). Therefore, the relationship between differentiation status and clinical behaviour is more complex than a simple stemness‐versus‐differentiation paradigm. One possible explanation is that increased *PSEN1* expression within the SHH α MB subtype identifies tumours that remain developmentally constrained and retain transcriptional features of cerebellar progenitor populations, whereas reduced *PSEN1* expression may permit transition toward alternative neuronal differentiation programmes associated with greater cellular plasticity and tumour fitness. In this model, a favourable prognostic association of *PSEN1* would not necessarily reflect a protective effect of stemness itself, but rather the preservation of a lineage‐restricted developmental state. This hypothesis is consistent with emerging evidence indicating that MB progression is influenced not only by proliferative capacity but also by the acquisition of distinct developmental and neuronal programmes (Ghasemi et al. 2024). Further studies integrating transcriptomic, proteomic and single‐cell analyses will be necessary to determine whether *PSEN1* actively regulates these cellular states or functions primarily as a marker of favourable tumour differentiation trajectories.

Importantly, *PSEN1* was not selectively or particularly highly expressed in SHH α tumours compared with other MB subtypes. Thus, the present findings should not be interpreted as indicating that *PSEN1* is an expression marker of SHH α MB. Rather, they suggest that the clinical significance of variation in *PSEN1* expression is context‐dependent, with higher *PSEN1* expression being associated with favourable outcome specifically within the SHH α molecular background. Gene expression levels and the biological or clinical significance of their variation across individual tumours represent distinct phenomena; a gene need not be preferentially expressed in a tumour subtype for its expression to identify biologically or clinically distinct states within that subtype. The transcriptional programmes associated with *PSEN1* in SHH α tumours further support the possibility that its prognostic association reflects the particular molecular context of these tumours. Nevertheless, the present data establish an association and do not demonstrate an SHH α‐specific functional role for *PSEN1*, which will require validation in experimental models that faithfully reproduce this molecular subtype.

Our co‐expression and GO analyses revealed an unexpected pattern of *PSEN1* co‐expression in SHH α. The analyses did not recover a canonical SHH programme or a classical neurodevelopmental programme. Instead, most of the enriched terms converge on themes including endoplasmic reticulum (ER), Golgi, membrane trafficking, proteostasis and RNA homeostasis. Specific signalling pathways among the enriched terms included regulation of the transforming growth factor‐β (TGF‐β) receptor pathway and the canonical WNT pathway. The BP enrichment indicated RNA stabilization, mRNA stabilization, regulation of mRNA stability and negative regulation of mRNA catabolism, whereas the CC analysis identified Hrd1 ubiquitin ligase complex. The findings may be interpreted as suggesting a cellular state dedicated to maintaining protein and transcript integrity. In other words, *PSEN1*‐high SHH α MB may exhibit enhanced capacity for transcript maintenance, protein quality control, ER‐associated degradation (ERAD) and trafficking of newly synthesized proteins. Regarding the developmental signalling pathways, TGF‐β and WNT have known developmental interactions with *PSEN1* biology (Sanadgol et al. 2023; Lee et al. 2024). Overall, the results raise the possibility that high *PSEN1* expression in SHH α MB is associated with a cellular programme featuring intracellular membrane trafficking, ER‐Golgi function, protein quality control and modulation of developmental signalling pathways, rather than with canonical SHH pathway activation itself.

The independent‐cohort analysis further supports the association of *PSEN1* with a reproducible transcriptional state in SHH α MB. *PSEN1* expression was not selectively increased in SHH α compared with the other SHH molecular subtypes, supporting the interpretation that its association with favourable outcome in SHH α is unlikely to reflect simply greater expression of *PSEN1* in this subtype. More importantly, the *PSEN1*‐associated gene set identified in Cavalli SHH α tumours showed striking concordance in an independent set of 50 SHH α tumours, so that all 22 evaluable genes were positively correlated with *PSEN1*, and 15 ranked among the 100 strongest genome‐wide *PSEN1* associations. This enrichment remained highly significant when evaluated against the genome‐wide correlation background and by random gene‐set permutation. These findings suggest that the association of *PSEN1* with SHH α MB reflects a reproducible transcriptional context rather than a cohort‐specific co‐expression pattern.

Analysis of two independent single‐nucleus transcriptomic datasets of the developing human cerebellum provided additional developmental context for the association between *PSEN1* expression and favourable outcome in SHH α MB. In the Aldinger et al. (2021) dataset, *PSEN1* was broadly expressed across cerebellar cell populations and was therefore not selectively associated with the granule lineage. Nevertheless, expression was clearly detectable in RL cells, GCPs and GNs. Donor‐level analysis did not demonstrate significant differences in *PSEN1* expression across the RL–GCP–GN trajectory, arguing against a simple model in which *PSEN1* marks a particular stage of granule cell differentiation.

Analysis of an independent developing human cerebellum dataset from Sepp et al. (2024) confirmed *PSEN1* expression in GCPs and across differentiating granule cell states. Together, these observations indicate that *PSEN1* is physiologically expressed throughout the developmental lineage relevant to SHH MB biology, although it is neither restricted to this lineage nor selectively enriched at a specific developmental state. The developmental data therefore do not establish a mechanism underlying the prognostic association observed in SHH α tumours, but demonstrate that this association occurs in the context of a gene normally expressed in the relevant cerebellar developmental populations.

Our analysis across different paediatric brain tumour types provides additional context for the association of *PSEN1* with MB biology. *PSEN1* was broadly expressed across the seven tumour entities examined, although expression levels differed significantly among tumour types. MB occupied an intermediate position, with lower *PSEN1* expression than high‐grade glioma/astrocytoma, low‐grade glioma/astrocytoma and pilocytic astrocytoma, higher expression than EPN and levels comparable to those observed in ATRT and diffuse midline glioma. Thus, the favourable prognostic association of higher *PSEN1* expression observed in SHH α MB does not appear to reflect unusually high or MB‐specific expression of the gene. Instead, these findings reinforce the notion that the biological significance of *PSEN1* expression is likely to be context‐dependent. Together with the developmental cerebellar analyses showing *PSEN1* expression across granule cell lineage states, the cross‐tumour comparison supports a model in which the prognostic significance of *PSEN1* in SHH α MB may relate more closely to the cellular and transcriptional state in which it is expressed than to its absolute expression level alone.

Several limitations of this study must be acknowledged. First, *PSEN1* transcription levels do not necessarily reflect protein abundance, γ‐secretase activity or functional PS1 signalling. Second, a possible biological significance of *PSEN1* expression in SHH α MB was inferred from transcriptomic and bioinformatic analyses rather than direct experimental measurements; therefore, the present study is purely correlational and cannot establish causality. Likewise, the co‐expression networks and enriched BP identified here should be considered hypothesis‐generating rather than evidence of pathway activation or functional involvement. Currently available MB cell lines do not faithfully reproduce the SHH α molecular context underlying the principal findings reported here, and experiments using appropriately characterized patient‐derived or other SHH α‐representative models will therefore be required to establish whether *PSEN1* plays a causal role in this MB subtype. Additional studies integrating single‐cell, proteomic, functional and mechanistic approaches will be necessary to determine whether *PSEN1* actively contributes to SHH α MB biology or primarily serves as a marker of underlying cellular states.

The potential value of *PSEN1* as a candidate prognostic marker should be considered in the context of the molecular heterogeneity of MB. Biomarkers that provide information across multiple MB groups, such as *SLFN11*, may offer broader clinical applicability (Nakata et al. 2023). In contrast, the association observed here appears to be restricted to the SHH α molecular context. However, subtype restriction does not necessarily preclude biological or potential clinical relevance, particularly in a disease that is increasingly classified and treated according to molecularly defined entities. Rather, *PSEN1* may represent a candidate marker of biological heterogeneity within SHH α MB. Given the limited number of SHH α cases available for survival analysis, however, the present findings should be considered exploratory and will require confirmation in larger independent cohorts before any clinical application can be proposed.

## 5 Conclusion

The present study uses bioinformatic approaches to identify a possible prognostic association for *PSEN1* in SHH α MB, with elevated expression associated with improved patient survival. Importantly, this association was restricted to a specific molecular subtype, reinforcing the concept that biologically meaningful prognostic factors in MB may be obscured when analyses are performed exclusively at the subgroup level. The *PSEN1*‐associated transcriptional pattern identified in SHH α MB was reproduced in an independent cohort, providing additional support for the robustness of this molecular association. Moreover, *PSEN1* was expressed across cell populations of the normal developing human cerebellum, including the granule cell lineage, and across diverse paediatric brain tumour types, with MB displaying intermediate expression levels. Together, these findings suggest that the association of *PSEN1* with favourable outcome in SHH α MB is unlikely to reflect simply selective or unusually high expression of *PSEN1* in this tumour context.

The *PSEN1* expression was also associated with a transcriptional programme enriched for intracellular membrane trafficking, ER and Golgi function, protein quality control, RNA homeostasis and developmental signalling pathways including TGF‐β and WNT. Combined with the known roles of *PSEN1* in neural progenitor biology and Notch signalling, the developmental cerebellar findings and independent transcriptional validation raise the possibility that *PSEN1* marks a developmentally constrained tumour state characterized by preserved cellular homeostasis and reduced malignant plasticity. Although the mechanistic basis of this association remains to be established, our findings identify *PSEN1* and some of its associated molecular networks as candidates for future functional investigation. More broadly, this study highlights how subtype‐specific integrated with developmental and cross‐tumour transcriptomic contexts can uncover unexpected biological relationships and generate new hypotheses regarding the developmental programmes that influence MB progression and patient outcome.

## Author Contributions

**Julia Vanini:** conceptualization, methodology, writing – original draft preparation, data analysis, writing – reviewing and editing. **Amanda Thomaz:** methodology, data analysis, investigation, writing – reviewing and editing. **Marina Müller Lupatini:** conceptualization, methodology, writing – reviewing and editing. **André T. Brunetto:** writing – original draft preparation, writing – reviewing and editing. **Caroline Brunetto de Farias:** methodology, writing – original draft preparation, writing – reviewing and editing. **Mariane Jaeger:** writing – original draft preparation, writing – reviewing and editing. **Rafael Roesler:** conceptualization, methodology, writing – original draft preparation, data analysis, investigation, supervision, writing – reviewing and editing.

## Funding

This work was supported by the National Council for Scientific and Technological Development (CNPq, MCTI, Brazil) grant numbers 304623/2025‐3 and 406484/2022‐8 (INCT BioOncoPed) to R.R. and the Children's Cancer Institute (ICI).

## Ethics Statement

This study was based exclusively on analyses of publicly available, de‐identified transcriptomic datasets and publicly accessible online bioinformatic resources. No human participants were recruited, no human tissue or biological specimens were collected or analysed, no identifiable patient information was accessed and no animal experiments were performed. Therefore, institutional ethics committee approval and informed consent were not required.

## Conflicts of Interest

The authors declare no conflicts of interest.

## Supporting information


**Table S1:** Analyses of association between high or low *PSEN1* gene expression levels and OS in patients with different subtypes of MB tumours using different statistical criteria.
**Table S2:** Comparisons of *PSEN1* expression levels among different molecular subgroups of MB.
**Table S3:** Comparisons of *PSEN1* expression levels among different molecular subtypes of MB.
**Table S4:** Genes showing strong or very strong co‐expression with *PSEN1* in SHH α MB tumours.
**Figure S1:** jdn70178‐sup‐0001‐Supporting_Information.docx. *PSEN1* expression visualized according to age group, survival status, histological subtype and metastatic status in SHH α MB tumours. The analysis was performed using the R2 Genomics Analysis and Visualization Platform (https://hgserver1.amc.nl/). Data were obtained from the Cavalli cohort (Cavalli et al. 2017); *n* = 65.
**Figure S2:** jdn70178‐sup‐0001‐Supporting_Information.docx. *PSEN1* expression across SHH MB molecular subtypes in an independent cohort. *PSEN1* expression was examined in an independent cohort of molecularly characterized SHH MB using the harmonized expression dataset reported by Arora et al. (2026) and molecular subtype annotations from Skowron et al. (2021). The analysis included 50 SHH α, 42 SHH β, 32 SHH γ and 72 SHH δ tumours. Differences among subtypes were assessed using the Kruskal–Wallis test (*p* = 0.041), followed by pairwise Wilcoxon rank‐sum tests with BH correction for multiple comparisons. Only the difference between SHH β and SHH γ remained significant after BH correction (adjusted *p* = 0.026).
**Figure S3:** Independent validation of the *PSEN1*‐associated transcriptional pattern in SHH α MB. Pearson correlations between *PSEN1* expression and the prespecified Cavalli‐derived *PSEN1*‐associated gene set were examined in an independent cohort of 50 SHH α tumours. Of the 26 genes identified as strongly positively correlated with *PSEN1* in the Cavalli cohort, 22 were represented in the independent expression matrix. All 22 genes showed positive correlations with *PSEN1*, with Pearson correlation coefficients ranging from 0.674 to 0.944 and a median correlation coefficient of 0.880.
**Figure S4:** Independent validation of *PSEN1* expression across developing human cerebellar RL/EGL populations. *PSEN1* expression was examined in the independent developing human cerebellum single‐nucleus RNA‐sequencing dataset of Sepp et al. (2024). Cells assigned by the authors to the RL/EGL lineage were grouped according to the original developmental‐state annotations. Dot position indicates mean *PSEN1* expression, and dot size indicates the percentage of nuclei with detectable *PSEN1* expression. *PSEN1* was detected across granule cell precursor (GCP) and multiple differentiating granule cell (GC) states, independently confirming its expression throughout the developing granule cell lineage. UBC, unipolar brush cell; UBCP, unipolar brush cell progenitor.

## Data Availability

The tumour dataset used in this study is available in the Gene Expression Omnibus (GEO) repository, accession numbers GSE202043, https://www.ncbi.nlm.nih.gov/geo/query/acc.cgi?acc=GSE202043, GSE85217, https://www.ncbi.nlm.nih.gov/geo/query/acc.cgi?acc=GSE85217, and GSE156053, https://www.ncbi.nlm.nih.gov/geo/query/acc.cgi?acc=GSE156053. The SHH MB RNA‐sequencing dataset described by Skowron et al. (2021) is available from the European Genome‐Phenome Archive (EGA) under accession number EGAD00001006305 (https://ega‐archive.org/datasets/EGAD00001006305). The integrated MB transcriptomic dataset described by Arora et al. (2026) was generated from publicly available RNA‐sequencing datasets deposited under accession numbers E‐MTAB‐6814 (https://www.ebi.ac.uk/biostudies/arrayexpress/studies/E‐MTAB‐6814), GSE109381 (https://www.ncbi.nlm.nih.gov/geo/query/acc.cgi?acc=GSE109381), EGAS00001002696 (https://ega‐archive.org/studies/EGAS00001002696), EGAS00001000254 (https://ega‐archive.org/studies/EGAS00001000254), EGAD00001006305 (https://ega‐archive.org/dacs/EGAC00001001002) and EGAS00001005826 (https://ega‐archive.org/studies/EGAS00001005826). The MB scRNA‐seq single cell dataset is available at the Pediatric Neuro‐oncology Cell Atlas (https://www.pneuroonccellatlas.org/). Single‐nucleus RNA‐sequencing data from the developing human cerebellum reported by Aldinger et al. (2021) were obtained through the CELLxGENE collection *Spatial and cell‐type transcriptional landscape of human cerebellar development* (https://cellxgene.cziscience.com/collections/1b014f39‐f202‐45ae‐bb7d‐9286bddd8d8b). The independent developing human cerebellum dataset reported by Sepp et al. (2024) was obtained from the human snRNA‐seq dataset in the CELLxGENE collection *Cellular development and evolution of the mammalian cerebellum* (https://cellxgene.cziscience.com/e/bab7432a‐5cfe‐45ea‐928c‐422d03c45cdd.cxg/). *PSEN1* expression across paediatric brain tumour types was examined using RNA‐sequencing data from the Open Pediatric Brain Tumor Atlas (OpenPBTA; https://github.com/AlexsLemonade/OpenPBTA‐analysis).

## References

[jdn70178-bib-0001] Aldinger, K. A. , Z. Thomson , I. G. Phelps , et al. 2021. “Spatial and Cell Type Transcriptional Landscape of Human Cerebellar Development.” Nature Neuroscience 24, no. 8: 1163–1175. 10.1038/s41593-021-00872-y. 34140698 PMC8338761

[jdn70178-bib-0002] Arber, C. , C. Lovejoy , L. Harris , et al. 2021. “Familial Alzheimer's Disease Mutations in PSEN1 Lead to Premature Human Stem Cell Neurogenesis.” Cell Reports 34, no. 2: 108615. 10.1016/j.celrep.2020.108615. 33440141 PMC7809623

[jdn70178-bib-0003] Arora, S. , N. Nuechterlein , M. Jensen , et al. 2026. “Integrated Transcriptomic Landscape of Medulloblastoma and Ependymoma Reveals Novel Tumor Subtype‐Specific Biology.” Neuro‐Oncology 28, no. 2: 505–519. 10.1093/neuonc/noaf251. 41159380 PMC12979040

[jdn70178-bib-0004] Bowman, R. L. , Q. Wang , A. Carro , R. G. Verhaak , and M. Squatrito . 2017. “GlioVis Data Portal for Visualization and Analysis of Brain Tumor Expression Datasets.” Neuro‐Oncology 19, no. 1: 139–141. 10.1093/neuonc/now247. 28031383 PMC5193031

[jdn70178-bib-0005] Cavalli, F. M. G. , M. Remke , L. Rampasek , et al. 2017. “Intertumoral Heterogeneity Within Medulloblastoma Subgroups.” Cancer Cell 31, no. 6: 737–754.e6. 10.1016/j.ccell.2017.05.005. 28609654 PMC6163053

[jdn70178-bib-0006] Cho, Y. J. , A. Tsherniak , P. Tamayo , et al. 2011. “Integrative Genomic Analysis of Medulloblastoma Identifies a Molecular Subgroup That Drives Poor Clinical Outcome.” Journal of Clinical Oncology 29, no. 11: 1424–1430. 10.1200/JCO.2010.28.5148. 21098324 PMC3082983

[jdn70178-bib-0007] Coelho, K. M. P. A. , M. Dalmolin , F. R. Pedack , et al. 2025. “Opposite Patterns of Association of TWIST1 Expression With Patient Survival in SHH and Group 4 Medulloblastoma.” Child's Nervous System 41, no. 1: 277. 10.1007/s00381-025-06913-2. 40936033

[jdn70178-bib-0008] Coltin, H. , L. Sundaresan , K. S. Smith , et al. 2021. “Subgroup and Subtype‐Specific Outcomes in Adult Medulloblastoma.” Acta Neuropathologica 142, no. 5: 859–871. 10.1007/s00401-021-02358-4. 34409497 PMC10723183

[jdn70178-bib-0009] de Araújo, M. A. , O. Malafaia , J. M. Ribas Filho , L. Fratini , R. Roesler , and G. R. Isolan . 2023. “Low Expression of the NRP1 Gene Is Associated With Shorter Overall Survival in Patients With Sonic Hedgehog and Group 3 Medulloblastoma.” International Journal of Molecular Sciences 24, no. 14: 11601. 10.3390/ijms241411601. 37511358 PMC10380701

[jdn70178-bib-0010] Falcón, P. , E. Fernández‐Caniulén , J. M. Zúñiga‐Hernández , et al. 2026. “The SHH/GLI Target Netrin‐1 Supports Self‐Renewal in Medulloblastoma Tumor Cells.” Cell Communication and Signaling. 10.1186/s12964-026-02993-y. PMC13587312 42298590

[jdn70178-bib-0011] Fratini, L. , M. G. S. Dalmolin , M. Sinigaglia , et al. 2023. “ZEB1 Is a Subgroup‐Specific Marker of Prognosis and Potential Drug Target in Medulloblastoma.” Neuromolecular Medicine 25, no. 1: 64–74. 10.1007/s12017-022-08716-z. 35716340

[jdn70178-bib-0012] Ghasemi, D. R. , K. Okonechnikov , A. Rademacher , et al. 2024. “Compartments in Medulloblastoma With Extensive Nodularity Are Connected Through Differentiation Along the Granular Precursor Lineage.” Nature Communications 15, no. 1: 269. 10.1038/s41467-023-44117-x. PMC10774372 38191550

[jdn70178-bib-0013] Gibson, P. , Y. Tong , G. Robinson , et al. 2010. “Subtypes of Medulloblastoma Have Distinct Developmental Origins.” Nature 468, no. 7327: 1095–1099. 10.1038/nature09587. 21150899 PMC3059767

[jdn70178-bib-0014] Gold, M. P. , W. Ong , A. M. Masteller , et al. 2024. “Developmental Basis of SHH Medulloblastoma Heterogeneity.” Nature Communications 15, no. 1: 270. 10.1038/s41467-023-44300-0. PMC10774283 38191555

[jdn70178-bib-0015] Handler, M. , X. Yang , and J. Shen . 2000. “Presenilin‐1 Regulates Neuronal Differentiation During Neurogenesis.” Development 127, no. 12: 2593–2606. 10.1242/dev.127.12.2593. 10821758

[jdn70178-bib-0016] Hovestadt, V. , K. S. Smith , L. Bihannic , et al. 2019. “Resolving Medulloblastoma Cellular Architecture by Single‐Cell Genomics.” Nature 572, no. 7767: 74–79. 10.1038/s41586-019-1434-6. 31341285 PMC6754173

[jdn70178-bib-0017] Juraschka, K. , and M. D. Taylor . 2019. “Medulloblastoma in the Age of Molecular Subgroups: A Review.” Journal of Neurosurgery: Pediatrics 24, no. 4: 353–363. 10.3171/2019.5.PEDS18381. 31574483

[jdn70178-bib-0018] Lee, C. Y. , D. Riffle , Y. Xiong , et al. 2024. “Characterizing Dysregulations via Cell‐Cell Communications in Alzheimer's Brains Using Single‐Cell Transcriptomes.” BMC Neuroscience 25, no. 1: 24. 10.1186/s12868-024-00867-y. 38741048 PMC11089696

[jdn70178-bib-0019] Louvi, A. , S. S. Sisodia , and E. A. Grove . 2004. “Presenilin 1 in Migration and Morphogenesis in the Central Nervous System.” Development 131, no. 13: 3093–3105. 10.1242/dev.01191. 15163631

[jdn70178-bib-0020] Monteiro, J. M. , M. Dalmolin , M. A. C. Fernandes , et al. 2024. “High Expression of GABAA Receptor β Subunit Genes Is Associated With Longer Overall Survival in Medulloblastoma.” Brain Sciences 14, no. 11: 1146. 10.3390/brainsci14111146. 39595908 PMC11592162

[jdn70178-bib-0021] Nakata, S. , J. Murai , M. Okada , et al. 2023. “Epigenetic Upregulation of Schlafen11 Renders WNT‐ and SHH‐Activated Medulloblastomas Sensitive to Cisplatin.” Neuro‐Oncology 25, no. 5: 899–912. 10.1093/neuonc/noac243. 36273330 PMC10158119

[jdn70178-bib-0022] Northcott, P. A. , I. Buchhalter , A. S. Morrissy , et al. 2017. “The Whole‐Genome Landscape of Medulloblastoma Subtypes.” Nature 547, no. 7663: 311–317. 10.1038/nature22973. 28726821 PMC5905700

[jdn70178-bib-0023] Northcott, P. A. , A. Korshunov , S. M. Pfister , and M. D. Taylor . 2012. “The Clinical Implications of Medulloblastoma Subgroups.” Nature Reviews Neurology 8, no. 6: 340–351. 10.1038/nrneurol.2012.78. 22565209

[jdn70178-bib-0024] Northcott, P. A. , G. W. Robinson , C. P. Kratz , et al. 2019. “Medulloblastoma.” Nature Reviews Disease Primers 5, no. 1: 11. 10.1038/s41572-019-0063-6. 30765705

[jdn70178-bib-0025] Oliver, T. G. , T. A. Read , J. D. Kessler , et al. 2005. “Loss of Patched and Disruption of Granule Cell Development in a Pre‐Neoplastic Stage of Medulloblastoma.” Development 132, no. 10: 2425–2439. 10.1242/dev.01793. 15843415

[jdn70178-bib-0026] Paganelli, A. R. , O. H. Ocaña , M. I. Prat , et al. 2001. “The Alzheimer‐Related Gene Presenilin‐1 Facilitates Sonic Hedgehog Expression in Xenopus Primary Neurogenesis.” Mechanisms of Development 4773, no. 1: 119–131. 10.1016/s0925-4773(01)00458-0. 11520668

[jdn70178-bib-0027] Riemondy, K. A. , S. Venkataraman , N. Willard , et al. 2022. “Neoplastic and Immune Single‐Cell Transcriptomics Define Subgroup‐Specific Intra‐Tumoral Heterogeneity of Childhood Medulloblastoma.” Neuro‐Oncology 24, no. 2: 273–286. 10.1093/neuonc/noab135. 34077540 PMC8804892

[jdn70178-bib-0028] Saciloto, B. , M. Dalmolin , J. C. Marcolin , et al. 2026. “Subgroup‐Specific Associations of GRIA Genes Encoding AMPA Glutamate Receptor Subunits With Patient Survival in Medulloblastoma.” Neuromolecular Medicine 28, no. 1: 36. 10.1007/s12017-026-08935-8. 42277445 PMC13260187

[jdn70178-bib-0029] Sanadgol, N. , J. Amini , C. Beyer , and A. Zendedel . 2023. “Presenilin‐1‐Derived Circular RNAs: Neglected Epigenetic Regulators With Various Functions in Alzheimer's Disease.” Biomolecules 13, no. 9: 1401. 10.3390/biom13091401. 37759801 PMC10527059

[jdn70178-bib-0030] Sepp, M. , K. Leiss , F. Murat , et al. 2024. “Cellular Development and Evolution of the Mammalian Cerebellum.” Nature 625, no. 7996: 788–796. 10.1038/s41586-023-06884-x. 38029793 PMC10808058

[jdn70178-bib-0031] Shapiro, J. A. , K. S. Gaonkar , S. J. Spielman , et al. 2023. “OpenPBTA: The Open Pediatric Brain Tumor Atlas.” Cell Genomics 3, no. 7: 100340. 10.1016/j.xgen.2023.100340. 37492101 PMC10363844

[jdn70178-bib-0032] Sherrington, R. , E. I. Rogaev , Y. Liang , et al. 1995. “Cloning of a Gene Bearing Missense Mutations in Early‐Onset Familial Alzheimer's Disease.” Nature 375, no. 6534: 754–760. 10.1038/375754a0. 7596406

[jdn70178-bib-0033] Skowron, P. , H. Farooq , F. M. G. Cavalli , et al. 2021. “The Transcriptional Landscape of Shh Medulloblastoma.” Nature Communications 12, no. 1: 1749. 10.1038/s41467-021-21883-0. PMC7979819 33741928

[jdn70178-bib-0034] Taylor, M. D. , P. A. Northcott , A. Korshunov , et al. 2012. “Molecular Subgroups of Medulloblastoma: The Current Consensus.” Acta Neuropathologica 123, no. 4: 465–472. 10.1007/s00401-011-0922-z. 22134537 PMC3306779

[jdn70178-bib-0035] Vladoiu, M. C. , I. El‐Hamamy , L. K. Donovan , et al. 2019. “Childhood Cerebellar Tumours Mirror Conserved Fetal Transcriptional Programs.” Nature 572, no. 7767: 67–73. 10.1038/s41586-019-1158-7. 31043743 PMC6675628

[jdn70178-bib-0036] Wallace, V. A. 1999. “Purkinje‐Cell‐Derived Sonic Hedgehog Regulates Granule Neuron Precursor Cell Proliferation in the Developing Mouse Cerebellum.” Current Biology 9, no. 8: 445–448. 10.1016/s0960-9822(99)80195-x. 10226030

[jdn70178-bib-0037] Wechsler‐Reya, R. J. , and M. P. Scott . 1999. “Control of Neuronal Precursor Proliferation in the Cerebellum by Sonic Hedgehog.” Neuron 22, no. 1: 103–114. 10.1016/s0896-6273(00)80682-0. 10027293

[jdn70178-bib-0038] Yang, Z. J. , T. Ellis , S. L. Markant , et al. 2008. “Medulloblastoma Can Be Initiated by Deletion of Patched in Lineage‐Restricted Progenitors or Stem Cells.” Cancer Cell 14, no. 2: 135–145. 10.1016/j.ccr.2008.07.003. 18691548 PMC2538687

